# Prevention and Co-Management of Breast Cancer-Related Osteoporosis Using Resveratrol

**DOI:** 10.3390/nu16050708

**Published:** 2024-02-29

**Authors:** Christine Meyer, Aranka Brockmueller, Constanze Buhrmann, Mehdi Shakibaei

**Affiliations:** 1 Chair of Vegetative Anatomy, Institute of Anatomy, Faculty of Medicine, LMU Munich, Pettenkoferstr. 11, 80336 Munich, Germany; christine.meyer@med.uni-muenchen.de (C.M.); aranka.brockmueller@med.uni-muenchen.de (A.B.); 2 Institute of Anatomy and Cell Biology, Faculty of Medicine, University of Augsburg, 86159 Augsburg, Germany; constanze.buhrmann@med.uni-augsburg.de

**Keywords:** resveratrol, breast cancer, osteoporosis, inflammation, epigenetics, bone metabolism

## Abstract

Breast cancer (BC) is currently one of the most common cancers in women worldwide with a rising tendency. Epigenetics, generally inherited variations in gene expression that occur independently of changes in DNA sequence, and their disruption could be one of the main causes of BC due to inflammatory processes often associated with different lifestyle habits. In particular, hormone therapies are often indicated for hormone-positive BC, which accounts for more than 50–80% of all BC subtypes. Although the cure rate in the early stage is more than 70%, serious negative side effects such as secondary osteoporosis (OP) due to induced estrogen deficiency and chemotherapy are increasingly reported. Approaches to the management of secondary OP in BC patients comprise adjunctive therapy with bisphosphonates, non-steroidal anti-inflammatory drugs (NSAIDs), and cortisone, which partially reduce bone resorption and musculoskeletal pain but which are not capable of stimulating the necessary intrinsic bone regeneration. Therefore, there is a great therapeutic need for novel multitarget treatment strategies for BC which hold back the risk of secondary OP. In this review, resveratrol, a multitargeting polyphenol that has been discussed as a phytoestrogen with anti-inflammatory and anti-tumor effects at the epigenetic level, is presented as a potential adjunct to both support BC therapy and prevent osteoporotic risks by positively promoting intrinsic regeneration. In this context, resveratrol is also known for its unique role as an epigenetic modifier in the regulation of essential signaling processes—both due to its catabolic effect on BC and its anabolic effect on bone tissue.

## 1. Introduction

Breast cancer (BC) is the most common cancer among women, with an estimated annual incidence of more than two million cases globally and with a rising tendency, making BC a global health concern [1]. Despite early detection and a variety of elaborated treatment strategies, in women, BC remains the second leading cause of death in developed countries [2]. However, survival rates have also increased for certain BC subtypes, particularly the early-stage hormone-sensitive BC subtype with overexpression of the estrogen α receptor, which responds to up to 70% to hormonal therapies such as estrogen receptor blockers or aromatase inhibitors that suppress estrogen synthesis [3,4]. Since estrogen receptors are not only found in breast tissue but also in other organs such as bones, with an essential anabolic role in the bone remodeling process, a temporary or long-term estrogen deficiency leads to serious side effects such as secondary osteoporosis (OP) [5,6]. Therefore, in addition to postmenopausal women, premenopausal women with BC also represent a high-risk group for the onset of OP as a serious side effect of BC therapy [5,7]. Furthermore, BC therapies, such as chemo- and radiotherapy, are cohesive with an increased risk of secondary OP due to increased inflammatory processes [6,8].

Various lifestyle habits, such as smoking, insufficient physical activity, and pro-inflammatory dietary habits, lead to epigenetic changes which positively correlate with the development of BC and OP [9]. Inflammatory processes are discussed as one of the primary causes of epigenetic changes that dysregulate a variety of cellular processes such as autophagy, which is essential for intact cell and tissue homeostasis [10]. In this regard, dysregulated autophagy in OP has been found in association with catabolic effects on the bone matrix, which is consequently suggested as a major dysregulated signaling process in OP [11]. In addition, the dysregulation of multiple other cellular processes such as apoptosis has been found to promote tumorigenesis as well as osteoporotic processes under a pro-inflammatory microenvironment characterized by increased expression of transcription factors such as nuclear factor-kappa B (NF-κB) and receptor activator of nuclear factor (NF)-κB ligand (RANKL) [12,13]. NF-κB and RANKL are involved in BC tumorigenesis, chemotherapy resistance, and survival rate [13], as well as in the dysregulation of the bone remodeling process by stimulating osteoclastogenesis and impeding osteogenesis [14,15,16].

Current conventional standard treatment for BC therapy-induced secondary OP commonly involves the use of bisphosphonates, which are monoclonal antibodies to RANKL, stabilizing a “status quo” in the bones by inhibiting bone resorption in a monotarget way without promoting anabolic effects on bone tissue [17]. Furthermore, non-steroidal anti-inflammatory drugs (NSAIDs) and cortisone, commonly used for musculoskeletal pain, impair intrinsic bone regeneration [18]. Therefore, there is an urgent need for new, clinically safe, and effective drugs that act on multiple targets by modulating inflammatory processes in accordance with anabolic effects on bone tissue and catabolic effects on BC cells.

A promising approach seems to be the strategy of prevention and co-therapy with phytopharmaceuticals, which are chemical substances produced by various plants that generally help them to build resistance against viral, bacterial, and fungal pathogens through their protective effects, without suppressing but rather promoting intrinsic cell regeneration pathways [19]. Polyphenols are an important group of phytopharmaceuticals associated with these properties and resveratrol has been one of the most intensively studied in recent times, particularly because of its attributed role with regard to longevity and its promising therapeutic potential, including cardioprotection and anti-cancer effects [20]. Sources of resveratrol include a variety of plants and fruits, such as red grapes, cranberries, mulberries, blueberries, jackfruit, peanuts, and eucalyptus, and it is generally considered to be clinically safe and non-toxic when consumed in moderate amounts in the diet [19]. As a bioactive polyphenol, resveratrol has a variety of multitarget effects, including anti-oxidative, anti-inflammatory, anti-carcinogenic, and immunomodulatory properties through the regulation of major inflammatory pathways by modulating the expression of the mentioned pro-inflammatory transcription factors as well as cytokines [19]. Furthermore, the phytoalexin is known to modulate estrogen receptor activity through its phytoestrogenic property, which is associated with anabolic effects on intrinsic bone regeneration and distinguishes resveratrol from other phytopharmaceuticals [21]. In addition, many resveratrol-related dose-dependent effects in vitro and in vivo show positive results at low doses and negative results at higher doses and can therefore be explained by a hormonal dose–response effect [22,23].

This review provides a comprehensive and robust analysis of the evidence supporting the use of resveratrol as a prophylactic and potential adjunct in the standard treatment of BC to support cancer therapy and to prevent or co-treat secondary OP by discussing the molecular mechanisms and benefits of using this phytopharmaceutical and its implications for clinical practice and future research.

## 2. Bone Health

Bone health is the foundation for an active and healthy lifestyle, as bones carry the points of origin and attachment of muscle tendons, protect internal organs, house bone marrow, are the largest repository of calcium and phosphate, serve as a reservoir for growth hormones, and also include the role of an endocrine organ by producing hormones such as fibroblast growth factor (FGF) 23 [24] and osteocalcin (OCN) [25].

Microstructurally, bone tissue is classified as a very dense, specialized form of connective tissue, consisting largely of an extracellular matrix (ECM) composed of 20 to 40% of organic matrix molecules and 60 to 70% of inorganic matrix components, together building up the structural framework and mechanical support for bone tissue [25]. The organic matrix, also referred as osteoid, represents the unmineralized matrix acting as a precursor to the mineralized matrix and is composed of fibers and an amorphous ground substance [25,26]. Type I collagen represents the major fiber in approximately 95% of the osteoid and provides tensile strength by forming fibrils [27,28].

Bone metabolism is based on the principle of constant remodeling through bone formation (osteogenesis) and bone resorption (osteolysis), which is primarily maintained by osteoblasts, osteoclasts, and osteocytes through cell–cell and cell–matrix communication, allowing them to adapt the ECM according to altering internal and external environmental conditions, illustrating that bone tissue is a metabolically extremely active structure [29].

**Figure 1:**
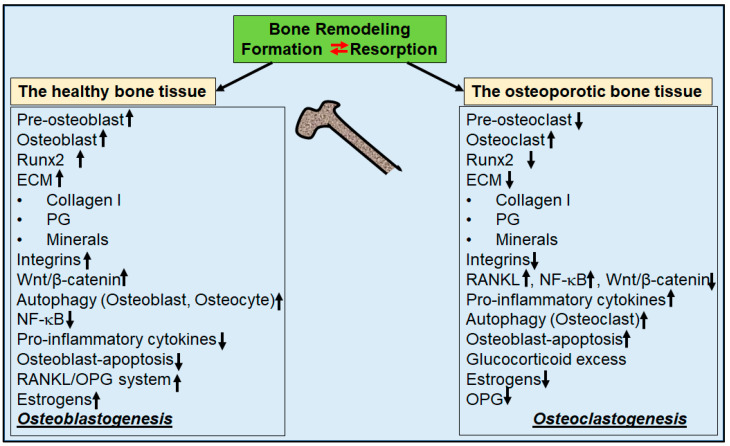
Schematic representation of bone properties at the level of active bone metabolism and osteoporosis. Abbreviations: ECM, extracellular matrix; Runx2, Runt-related transcription factor 2; RANKL, receptor activator of nuclear factor (NF)-kB (RANK)/receptor activator of nuclear factor (NF)-κB ligand; PG, proteoglycan; OPG, osteoprotegerin; NF-κB, nuclear factor-kappa B. The up arrow (↑) indicates activation/increase/high regulation and the down arrow (↓) indicates decrease/decrease/regulation/suppression.

The major builder of organic ECM molecules are osteoblasts, derived from mesenchymal stem cells (MSC) [30], highlighting the tremendous importance of maintaining osteoblast vitality for the formation of an intact ECM. Furthermore, this process has been shown to be stimulated by several osteogenic transcription factors such as Runt-related transcription factor 2 (Runx2), leading to an increased number of pre-osteoblasts [31]. Runx2 is an early osteogenic transcription factor for osteogenesis involved in MSC differentiation through the silent information regulator sirtuin 1(Sirt-1)/Runx2 axis [31] via the canonical Wingless (Wnt) signaling pathway [32]. In addition, it is well known that osteoblasts express various molecules, such as RANKL and osteoprotegerin (OPG), depending on their extracellular microenvironment, which enable them to regulate their counterparts, osteoclasts [33] (Figure 1).

Osteoclasts are derived from several mononuclear precursors of the monocyte/macrophage lineage of the hematopoietic system and play an essential role in bone resorption [34]. Their activity is known to be regulated by many signaling processes, including the RANKL/OPG ratio, with increased expression of RANKL promoting osteoclastogenesis and increased OPG counteracting it [33] (Figure 1). Therefore, a well-balanced RANKL/OPG ratio is one of the hallmarks of an intact bone metabolism [33]. RANKL binds to its RANK receptor on the osteoclast surface, activating central signaling pathways such as the NF-κB pathway, which stimulate osteoclasts to synthesize and secrete catabolic enzymes such as matrix metalloproteinase (MMP) that resorb bone ECM [33]. In contrast, OPG binds to RANKL in the form of a decoy receptor, thereby preventing RANKL from binding to its RANK receptor, leading to a reduction in bone resorption [33].

The third major key player in maintaining bone homeostasis are osteocytes within the mineralized matrix, which develop from osteoblasts and are about ten times more abundant [35]. Osteocytes are indirectly involved in the process of bone remodeling through their ability to modulate the activity of both osteoblasts and osteoclasts by secreting and expressing appropriate signaling molecules, such as sclerostin, which correlates with the suppression of the Wnt signaling pathway [36,37]. Due to their mechanosensitive property, allowing them to sense and respond to changes in external mechanical loading, they are able to exchange information with other osteocytes via their long cell processes, which is essential for ECM adaptation [38,39,40,41]. Moreover, all three cell types, osteoblasts, osteoclasts, and osteocytes, have contact with ECM molecules through a large number of specific integral membrane receptor proteins, the so-called integrins, which are located both on the surface of bone cells and on ECM molecules, such as collagen type I and fibronectin [42,43]

Estrogen is a key hormone for intact bone metabolism, which has been found to influence the expression of integrins involved in the mechanosensation of osteocytes, leading to modified osteoclastogenic paracrine signaling [44] (Figure 1). Moreover, hormones such as estrogen [45] and also conditions such as fasting [46] correlate with the inhibition of autophagy regulators, i.e., the mammalian target of rapamycin (mTOR)/phosphatidylinositol 3-kinase (PI3K)/protein kinase B (Akt) signaling pathway, thereby stimulating the autophagy process in osteoblasts in a positive physiological manner. With regard to estrogen and bone health, there is evidence that estrogen may increase the expression of autophagy proteins including Beclin-1 and LC3 in association with up-regulation of Sirt-1 in osteoblasts and osteocytes [47].

## 3. Epigenetic Changes as Key Driving Factor of Breast Cancer and Osteoporosis Development

The field of epigenetics is generally focused on hereditary deviations in gene expression that occur independently of changes in the DNA sequence. Numerous environmental impacts such as dietary components, physical activity, infections, toxins, and other agents modify the genome in beneficial or harmful ways. Indeed, pro-inflammatory lifestyle habits have been shown to induce epigenetic modifications, such as DNA methylations, histone acethylation, phosphorylation, methylation, and altered miRNA profiles, providing opportunities for the prevention and adjunctive treatment of chronic inflammatory diseases such as BC and OP [48,49]. Moreover, chronic inflammation is widely recognized as a major cause of BC and OP [50].

### 3.1. Inflammation as a Fundamental Cause of the Development of Breast Cancer and Osteoporosis

**Figure 2:**
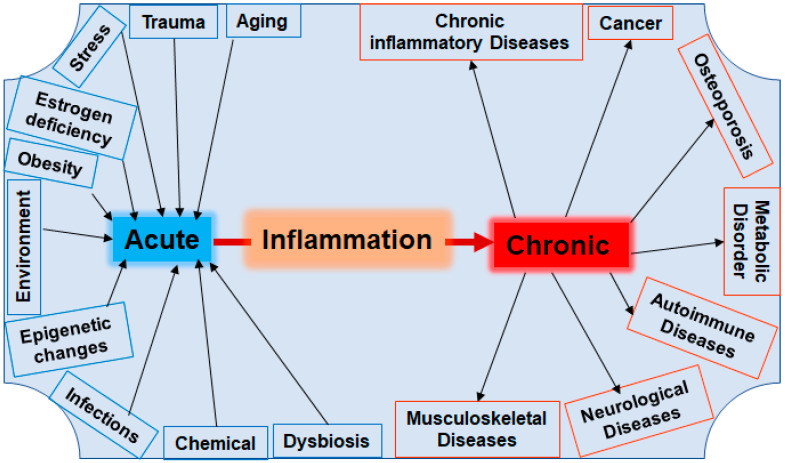
Several causes of acute inflammation, transition to chronic inflammation, and diseases associated with chronic inflammation.

Acute inflammation is the body’s physiological response to pathogens, injuries, or tissue dysfunction which is essential for the activation of the intrinsic regenerative process based on a highly complex and multifaceted interplay of pro-inflammatory enzymes, cytokines, and immune cells [50]. An intact immune system guarantees that acute inflammation subsides after a few days to a few weeks, during which the elevated levels of pro-inflammatory mediators return to baseline levels [50]. In contrast, persistent inflammation over months, years, or decades leads to a constitutive activation of pro-inflammatory signaling pathways, including the NF-κB pathway, associated with many chronic diseases, including BC and OP [50] (Figure 2, Table 1 and Table 2).

It is well established that various lifestyle factors, such as hypercaloric dietary habits with a high proportion of omega-6 polyunsaturated fatty acids, simple carbohydrates, low-fiber foods, and food additives, noxious and harmful substances including nicotine and alcohol, and insufficient or too much physical exercise and long-term stressors all promote chronic inflammatory processes, leading to significant metabolic changes, including intestinal dysbiosis [51,52,53] (Figure 2). Therefore, many of these factors are clinically categorized as risk factors for inflammatory chronic diseases, including BC and OP. Furthermore, there are endogenous risk factors such as the aging process correlated with deficiencies in reproductive hormones, including postmenopausal estrogen deficiency [54] (Figure 2).

**Figure 3:**
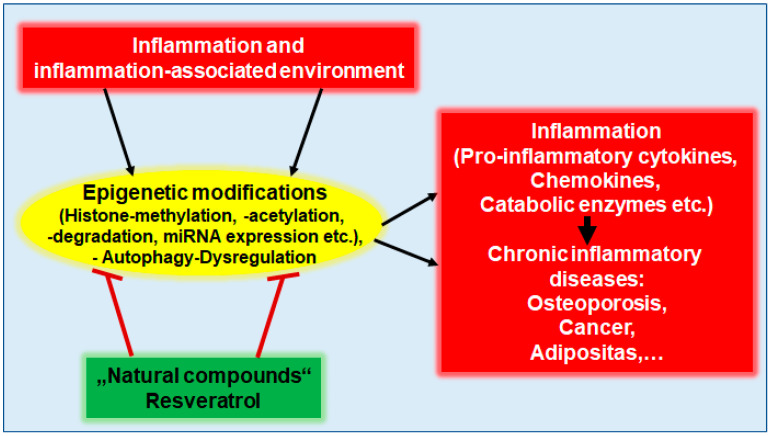
Epigenetic control of inflammation-related disorders by natural compounds.

According to current knowledge, dysregulations of the NF-κB, RANKL/RANK, and Wnt pathways are among the most important pro-inflammatory signaling pathways involved in BC and OP development at the epigenetic level [12,55,56] (Figure 3). Interestingly, anti-inflammatory agents such as dietary polyphenols have been found to reverse the constitutive activation of NF-κB through the modulation of epigenetic enzymes such as histone deacetylases, including Sirt-1 [16,57]. Therefore, a balanced diet rich in polyphenols and other anti-oxidants and anti-inflammatories can help maintain physiological tissue metabolism by stabilizing the individual epigenome in an anti-inflammatory manner, whereas a pro-inflammatory environment may be associated with genomic destabilization, which is the case with cancer stem cells [58] (Figure 3).

#### 3.1.1. Breast Cancer

BC is a multifactorial malignant disease with dysregulated cell growth and the potential ability of BC cells to infiltrate surrounding tissues [59]. Up to 80% of all BC cases show a hormone-dependent BC subtype [60,61]. Genetic factors play a minor role in most primary cancers, whereas epigenetic changes due to chronic inflammatory processes are implicated as a major cause of 90–95% of all cancers, including BC [62] (Figure 3). This fact is supported by clinical evidence showing that the development of both sporadic BC and hereditary BC is linked to epigenetic changes such as hypermethylation patterns, as familiar early-onset breast cancer gene (BRCA)-1 and BRCA-2 mutation carriers show different incidences of BC development depending on individual lifestyle factors such as smoking and the level of physical activity [63,64] (Figure 2 and Figure 3).

The close link between inflammation and tumorigenesis is supported by a large body of clinical data [65], which has also been reported for BC tumorigenesis [66,67,68,69,70,71] (Table 1). Tumorigenesis refers to the development and proliferation of a dynamic process with three stages, initiation, progression, and metastasis, involving cellular processes such as transformation, invasion, and angiogenesis [72].

Recent evidence suggests that pro-inflammatory cytokines combined with a loss of p53 in BC lead to an increased expression of Wnt ligands, thereby increasing the risk of metastasis [73]. Moreover, an increased level of soluble RANKL has been reported with an increased risk of estrogen-positive BC [71] leading to mammary epithelial proliferation and carcinogenesis [74]. This is in accordance with the finding that genetic inactivation of RANKL in mammary epithelial cells leads to a reduced incidence and delayed onset of BC [75].

Another inflammatory signaling pathway in BC is the Janus kinase (JAK) 2/signal transducer and activator protein (STAT) 3 signaling pathway, associated with poor BC outcomes due to increased risks of metastasis and therapy resistance [76]. This is in line with other data demonstrating that STAT3 stimulates cytokines in BC and that it has been associated with poor prognosis [77]. Furthermore, the pro-inflammatory VEGF seems to be another important key player in the tumorigenesis of BC [78].

This is consistent with the finding that in up to 70% of BC cases, a dysregulation of cellular processes such as autophagy has been reported to be associated with a reduced expression of autophagy-promoting proteins due to aberrant DNA methylation, leading to increased therapy resistance [79,80] (Figure 3). In order to specifically prevent tumorigenesis or reduce resistance to therapy, the TME (tumor microenvironment) therefore needs to be targeted, ideally with multitarget substances that attenuate the chronic inflammatory cascade at the epigenetic level [81,82]. An overview of other inflammatory cytokines and signaling pathways in association with BC is presented in Table 1.

**Table 1:** Pro-inflammatory cytokines and their association with breast cancer.

Pro-Inflammatory Cytokine	Study Concept	Influenced Signaling Pathways	Major Results	Reference
**TNF-α**	In vitro, 31EG4-2A4 cells	MMP-9, β1-integrin	Promotion of BC cell development, proliferation, and migration.	[83]
	In vitro, MCF10A cells	MMP-9, Smad, Ras, PI3k, TGF-β, HGF, EGF	Triggering of multipath crosstalk between stromal and BC cells.	[84]
	In vitro, MCF10A cells	NF-κB, Twist1	Induction of EMT and CSC development. Promotion of inflammation and BC metastasis.	[85]
	In vivo, NOD/SCID mice	VCAM-1, Ki67, Twist1, vimentin, slug	Promotion of BC proliferation, EMT, angiogenesis, and metastasis.	[86]
	*Clinical trial*, healthy humans and BC patients	IL-1β, inflammatory chemokines	Increase in EMT, BC progression, malignancy, and relapse.	[66]
	*Clinical trial*, healthy humans and BC patients	IL-6, IL-8	Correlation with BC stage, metastasis, and ER/HER2 expression.	[67]
**RANKL**	In vitro, BC cells	STAT3	Initiating autophagy and mediating chemoresistance of BC cells.	[87]
	In vivo, BC mice	Cyclin D1	Induction of BC promoting proliferation in mammary epithelia.	[74]
	*Clinical trial*, BC patients	OPG	Increasing the risk of ER-positive BC.	[71]
**IL-1**	In vitro, MDA-MB-231, MCF7 cells	p62, SOX9	Initiation of vital signaling cascades in BC cells.	[88]
**IL-1α/IL-1β**	*Clinical trial*, BC patients	IL-1α, IL-1β, IL-8	Promotion of pro-inflammatory TME, BC growth, and metastasis.	[68]
**IL-1β**	*Clinical trial*, healthy humans and BC patients	TNF-α, inflammatory chemokines	Increase in BC progression, malignancy, and relapse.	[66]
**IL-6**	In vitro, MCF-7, MDA-MB-468 cells	NF-κB, KPNA2, IL-8, IL-17	Stimulating of inflammation-based BC exacerbation.	[89]
**IL-6/IL-8**	*Clinical trial*, healthy humans and BC patients	TNF-α, IL-6, IL-8	Relation to BC aggressiveness and serving as prognostic biomarker.	[67]
**IL-22**	In vivo, C57/B6 mice	Twist1, Zeb-1, slug, snail	Up-regulation of EMT causing aggressiveness of BC in all stages.	[90]
**IL-30**	In vitro, BC cells In vivo, mice	IL-6, KISS1, STAT1, STAT3	Reinforcement of inflammation, vascularization, migration, and BC tumor growth.	[91]
**IL-33**	In vivo, BALB/c mice	IL-10, IL-13, ST2	Suppression of immune defenses and acceleration of BC progress.	[92]
**IFN-γ**	In vitro, BT-549 cells	JAK1, STAT1, IRF1	Suppression of immunoregulation and enabling of pro-inflammatory BC cell growth.	[93]
	*Clinical trial*, BC patients	PCNA	Increasing BC malignancy and optical tumor density.	[69]
**TGF-β**	In vitro, MDA-MB-231, T47D cells	EGFR, Smad3, ERK/Sp1	Up-regulation of BC cell proliferation, migration, and invasion.	[94]
	*Clinical trial*, BC patients	TNF-α, ER	Promotion of lymph node metastasis and serving as BC relapse prognostic marker.	[70]

Abbreviations: BC—breast cancer, CSC—cancer stem cell, EGF—epidermal growth factor, EGFR—epidermal growth factor receptor, EMT—epithelial–mesenchymal transition, ER—estrogen receptor, ERK—extracellular-signal regulated kinase, HER—human epidermal growth factor receptor, HGF—hepatocyte growth factor, IFN—interferon, IL—interleukin, IRF—interferon regulatory factor, JAK—Janus kinase, Ki67—Kiel antigen 67, KPNA2—karyopherin α-2, MMP—matrix metalloproteinase, NF-κB—nuclear factor kappa-light-chain-enhancer of activated B cells, OPG—osteoprotegerin, PCNA—proliferating cell nuclear antigen, PI3k—phosphoinositide 3-kinase, RANKL—receptor activator of NF-κB ligand, Ras—rat sarcoma, SOX9—SRY-related homeobox gene 9, STAT—signal transducer and activator protein, TGF—transforming growth factor, TME—tumor microenvironment, TNF—tumor necrosis factor, VCAM—vascular cell adhesion molecule, Zeb-1—Zinc finger E-box-binding homeobox.

#### 3.1.2. Osteoporosis

**Figure 4:**
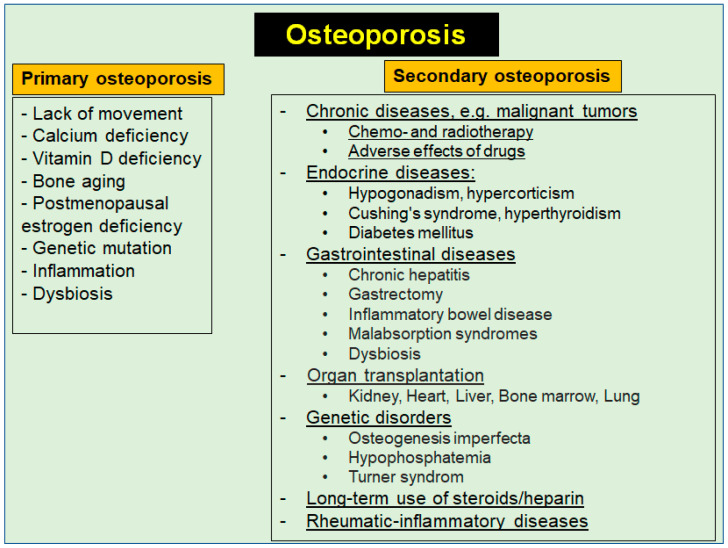
Promoting factors for primary and secondary osteoporosis.

Primary OP is a normal human aging process that can result from aging bones, lack of exercise, inflammation, calcium or vitamin D deficiency, postmenopausal estrogen deficiency, or when genetic mutations occur [95] (Figure 4). In contrast, the term secondary OP refers to bone loss due to inflammation and a number of defined clinical chronic diseases, including cancer, endocrine disorders, chronic malnutrition, autoimmune and genetic disorders (e.g., Turner syndrome), changes in the gut microbiota (dysbiosis), prolonged immobilization, as well as due to long-term drug therapy and adverse effects of drugs, including BC therapy [95,96] (Figure 4). It is therefore of great clinical importance to first clarify the reasons for bone loss before deciding on therapy.

In both forms of OP, there is a significant loss of bone mass, leading to an increased risk of fragility fractures which are associated with a high burden of musculoskeletal pain and reduced quality of life [5]. Considering the many underlying primary diseases and conditions of secondary OP, the complex problem of this disease, which is not yet curable, becomes evident [97] (Figure 4). The often-cited prevalence of more than 200 million affected people worldwide is likely to be far higher, also considering increasing life expectancy, with associated estrogen deficiency in postmenopausal women [98]. This hypothesis is supported by recent data showing a tremendous gap in the diagnosis and treatment of OP [98]. However, it is important to emphasize that early diagnosis of beginning or predictable bone loss offers preventive or adjuvant treatment options counteracting critical bone resorption processes.

Microstructurally, decreased bone mass in OP results from a dysregulation of bone remodeling in favor of bone resorption via increased osteoclastogenesis and degradation of ECM proteins, including collagen type I and proteoglycans while bone formation is impaired [99] (Figure 1). There is strong evidence that chronic inflammatory processes play a major role in the dysregulated bone remodeling process in OP, evidenced by clinical findings of increased pro-inflammatory interleukins in OP patients [100,101,102,103] (Table 2). Furthermore, it has been reported that pro-inflammatory cytokines induce epigenetic changes, including altered miRNA profiles in OP, as in postmenopausal OP, about 331 miRNAs have been found to have altered expression patterns, with nearly 63% of miRNAs down-regulated and the other nearly 37% up-regulated, involving about 155 different genes [104]. In this regard, clinically, it has been found that overexpression of specific miRNAs impairs bone formation by targeting Osteoblast-specific transcription factor Osterix (Osx) and Runx2 [105,106]. Regarding Runx2, histone deacetylases such as Sirt-1 have been reported as integral targets for osteogenic differentiation of MSC [31]. Furthermore, there is substantial in vitro and in vivo evidence that pro-inflammatory cytokines suppress specific miRNAs, leading to the inhibition of osteogenic differentiation of MSC in estrogen deficiency-induced OP [107] (Table 2), which is supported by evidence that cytokines reduce bone ECM, including a decrease in β1-integrin and Runx2 [30] (Table 2). The decreased expression of integrin receptors has been found to activate cellular programs such as apoptosis due to the impairment of intercellular communication in osteocytes [108]. Moreover, cytokines have been reported to increase Fas-mediated apoptosis in osteoblasts [109] and to suppress Wnt signaling [110] by stimulating the expression of Wnt antagonists such as sclerostin and Dickkopf-related protein 1 (DKK1) in osteocytes and osteoblasts [111,112]. These have further been demonstrated in association with increased osteoclastogenesis via the NF-κB, RANKL/RANK/OPG, PI3K/Akt, and c-Jun *N*-terminal kinase (JNK) signaling pathways [15,113,114,115,116,117,118] (Table 2).

**Table 2:** Pro-inflammatory cytokines and their association with osteoporosis.

Pro-Inflammatory Cytokine	Study Concept	Signaling Pathway	Major Results	Reference
**TNF-α**	In vitro, human osteoblasts	Fas	Enhancement of Fas-mediated apoptosis; Fas expression.	[109]
	In vitro, human MSCs	ERK, JNK	Inhibition of osteogenic differentiation of MSCs by increasing P2Y receptor expression in estrogen deficiency-related OP.	[119]
	In vitro, primary bone marrow cells In vivo, OVX mice	JNK	Elevation of semaphorin3D expression is a contributing factor to OP caused by estrogen deficiency. Induction of RANKL-promoted osteoclast differentiation.	[113]
	In vitro, BMHSCs In vivo, OVX mice	PI3k/Akt	Up-regulation of P2Y purinoceptor 2 receptor expression, promotion of BMHSCs to differentiate into osteoclasts, and enhanced bone resorption.	[114]
	In vitro, RAW264.7 cells In vivo, clinical trials, OP patients	NF-κB, PI3k/Akt	Synergistically enhances RANKL-promoted osteoclast proliferation, contributing to OP in postmenopausal women.	[115]
	In vitro, human MSCs In vivo, mice	FGF and ERK-MAPK	Suppression of miR-21, which represses its target gene Spry1, inhibited osteogenic MSCs differentiation in estrogen deficiency-induced OP. Blocking TNF-α in OVX mice promoted bone formation by activating miR-21-Spry1 axis.	[107]
	In vitro, osteoblasts In vivo, mice	NF-κB, MAPK	Up-regulation of RANKL mRNA, TRAP-positive osteoblasts, and osteoclastogenesis.	[15]
	In vitro, osteoblast-like osteosarcoma cells	NF-κB	Up-regulation of cytokines (IL-6) and cell adhesion molecules (ICAM-1); promotion of bone resorption and inflammation.	[120]
**TNF-β**	In vitro, MSCs, osteoblasts	NF-κB, Sirt-1	Down-regulation of osteogenic differentiation of MSC; suppression of bone ECM, β1-Integrin, and Runx2.	[30]
**IL-1β**	In vitro, human osteoblasts	Fas	Enhancement of apoptosis of osteoblasts.	[109]
	In vivo, mice	IGF	Up-regulation of inducible nitric oxide synthase, IGF2, and chemokines (CX3CL1 and CXCL7). Enhancement of osteoclastogenesis.	[121]
	In vitro, bone marrow cells	NFATc1, c-Fos	Up-regulation of RANKL and osteoclastogenesis.	[122]
	*Clinical trial*, OP patients	IL-1β	In postmenopausal females, OP is related to IL-1β (-511C/T) polymorphism.	[102]
	*Clinical trial*, healthy humans and OP patients	IL-1β	A substantial negative reciprocal relationship between osteocalcin and cytokine IL-1β in healthy women and women with OP.	[103]
	In vitro, MLO-Y4 osteocytes	RANKL/RANK/OPG	IL-1β promotes osteoclastogenesis by modulating RANKL/OPG gene expression through osteocytes.	[116]
	In vitro, human osteoblastic cells	OPG-L	Induction of osteoclastogenesis by promoting OPG ligand expression.	[117]
	In vitro, bone marrow and Raw264.7 macrophages In vivo, OVX mice	NF-κB, RANKL	Enhancement of osteoclastogenesis in osteoclast-linked OP.	[118]
**IL-6**	*Clinical trial*, OP patients	STAT3	IL-6 in serum is an indicator of postmenopausal OP. Induction of osteoclastogenesis.	[101]
	In vitro, MC3T3-E1 cells and primary murine calvarial osteoblasts	SHP2/MEK2/ERK, SHP2/PI3k/Akt2, JAK/STAT3	IL-6 inhibits osteoblast differentiation via the SHP2/MEK2/ERK and SHP2/PI3k/Akt2 pathways, whereas it acts positively via JAK/STAT3.	[123]
	*Clinical trial*, OP patients	sgp130	The biological activity of IL-6 may increase with age and potentially influence age-related OP.	[100]

Abbreviations: Akt—protein kinase B, BMHSC—bone marrow hematopoietic stem cell, CXCL—CXC motif ligand, ERK—extracellular-signal regulated kinase, Fas—apoptosis antigen 1, FGF—fibroblast growth factor, ICAM-1—intercellular adhesion molecule 1, IGF—insulin-like growth factor, IL—interleukin, JNK—c-Jun *N*-terminal kinase, MAPK—mitogen-activated protein kinase, MEK—mitogen-activated protein kinase kinase, MSC—mesenchymal stem cell, NF-κB—nuclear factor kappa-light-chain-enhancer of activated B cells, OP—osteoporosis, OPG—osteoprotegerin, OVX—ovariectomized, P2Y—purinoceptor 2, PI3k—phosphoinositide 3-kinase, RANKL—receptor activator of NF-κB ligand, Runx2—Runt-related transcription factor 2, sgp130—soluble glycoprotein 130, SHP2—Src homology-2 domain-containing protein tyrosine phosphatase-2, Sirt—sirtuin, STAT—signal transducer and activator protein, TNF—tumor necrosis factor.

#### 3.1.3. Functional Signaling Interaction between Breast Cancer and Bone Cells

**Figure 5:**
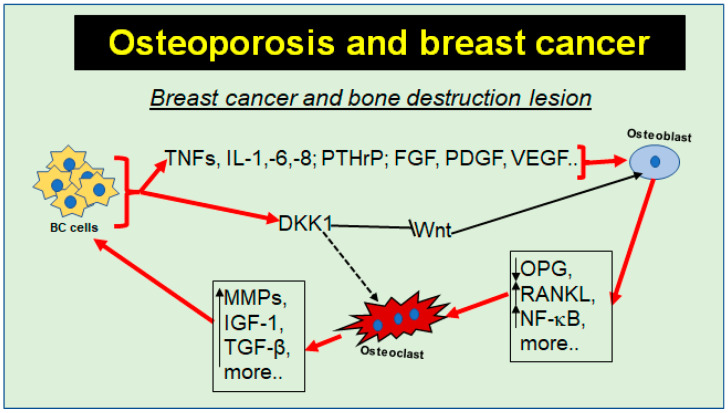
Complex development of secondary osteoporosis and osteolysis in breast cancer. Mammary cancer cells produce several factors, including various ILs, TNFs, vascular endothelial growth factor (VEGF), and parathyroid hormone-related protein (PTHrP), which act on osteoblasts and/or osteoclasts. In osteoblasts, there is an increase in the release of receptor activator of nuclear factor-κB ligand (RANKL), an osteoclast differentiation factor, and a decrease in the formation of osteoprotegerin (OPG). BC cells further synthesize the Wnt antagonist Dickkopf-related protein 1 (DKK1), inhibiting intrinsic bone regeneration and indirectly enhancing osteoclast-activating signaling pathways such as NF-κB and RANK/RANKL, which may contribute to osteolysis in BC patients. Abbreviations: PDGF—platelet-derived growth factor, MMP—matrix metalloproteinase, BC—breast cancer, TNF—tumor necrosis factor, IL—interleukin, IGF—insulin-like growth factor, TGF—transforming growth factor, NF—κB-nuclear factor kappa-light-chain-enhancer of activated B cells, Wnt—Wingless. The up arrow (↑) indicates activation/increase/high regulation and the down arrow (↓) indicates decrease/decrease/regulation/suppression.

As shown in the previous subchapters, inflammatory pathways such as NF-κB and RANK/RANKL/OPG play an integral role in both BC and OP and provide molecular evidence for a link between OP and BC, as they are known to promote both tumorigenesis and bone resorption [124]. In this regard, stromal cells in BC have been found to secrete RANKL and M-CSF, associated with increased osteoclastogenesis [13]. Moreover, the ability of BC cells to produce pro-inflammatory cytokines as well as hormones, growth factors, and VEGF has been reported to stimulate further tumorigenesis [125] and, synergistically, osteoclastogenesis (Figure 5). Additionally, osteoclasts have been shown to produce pro-inflammatory mediators such as TGF-β, MMPs, and growth hormones such as insulin-like growth factor (IGF-1), thereby reinforcing tumorigenesis [126] (Figure 5). These common mechanisms are also referred to as crosstalk between BC and bone cells, which promotes both TME and an osteoporotic microenvironment [126]. Another parallel between OP and BC is provided by the dysregulation of the Wnt pathway, which may be down-regulated by BC cells through synthesis and secretion of the Wnt antagonist DKK-1 [127], thereby inhibiting intrinsic bone regeneration and indirectly enhancing osteoclast-activating pathways such as NF-κB and RANK/RANKL, contributing to osteolysis in BC patients [128] (Figure 5).

### 3.2. Conventional Breast Cancer Treatment-Induced Secondary Osteoporosis

BC is commonly associated with skeletal morbidity, including secondary OP fractures [129], which might also be explained by the overlap of the inflammatory and epigenetic modifications, as highlighted in the previous subchapters. BC treatment-induced bone loss is a complex, multifactorial process, involving several factors, including the type of BC, the stage of the tumor, as well as the patient’s medical condition and the type of BC therapy [130].

However, approximately 70–80% of patients with early-stage, non-metastatic BC are curable [4] and according to the German Gynecological Oncology Group, the 5-year survival rate for early-stage BC without invasion or spread to other tissues is around 96% in European countries [131], which is broadly in accordance with the 5-year relative survival statistics from the US [2]. These data demonstrate the tremendous positive impact of early diagnosis and effective treatment options. Nevertheless, it is crucial to evaluate potential side effects associated with BC therapies such as surgical oophorectomy, chemotherapy-induced ovarian failure, and anti-estrogenic hormone therapies, including aromatase inhibitors, on the maintenance of a high quality of life for cancer survivors [132,133].

The high complexity of therapeutic management in BC is emphasized by the high variability between different patients and intra-tumoral heterogeneity within the same patient [59]. However, in about 80% of all BC cases, hormonal therapies are among the most commonly used treatment strategies, including selective estrogen receptor modulators and aromatase inhibitors [60,61]. These drugs target the estrogen receptor pathway to suppress the synthesis of estrogen or its activity, thereby also suppressing bone formation and remodeling processes, leading to secondary OP [134] (Figure 4). According to recent data, about 70–80% of all BC patients undergo adjuvant endocrine therapy for at least 5 years [135], and there is evidence that fragility fractures occur within less than 5 years of being started on aromatase inhibitors, illustrating the rapid progression of bone loss [136], as confirmed by a recent meta-analysis study [60].

Likewise, common chemotherapeutic agents have been found in correlation with enhanced bone resorption in premenopausal BC patients [137], which is consistent with findings from a longitudinal cohort study (N = 92,431) [133].

It is important to note that there are many long-term medical issues besides BC-induced secondary OP that are associated with estrogen deficiency, such as dementia, cardiovascular disease, and negative effects on mood and well-being, highlighting the multidimensional challenge in BC patients [138]. This issue is exacerbated by the frequent use of various medications such as anti-depressants and NSAIDS or other pain medications in BC patients [138,139], as these drugs have been found in association with increased bone resorption in the long term [140].

Although the onset of bone loss is undiagnosed in many BC patients, there are also an increasing number of diagnosed cases that integrate adjuvant therapy with monotarget anti-resorptive agents in BC patients [135]. Among the most commonly prescribed adjuvant treatment options are bisphosphonates, which are monoclonal antibodies against RANKL associated with reduced bone resorption [135] as well as pro-apoptotic effects on BC cells. However, bisphosphonates do not solve the fundamental problem of reduced bone formation as they primarily monotarget bone resorption and do not stimulate intrinsic bone regeneration, although periosteal bone formation is not inhibited by bisphosphonates [141]. Therefore, particularly for BC patients with treatment-induced bone loss, there is a need for novel multitarget adjuvant agents besides bisphosphonates that stimulate the intrinsic bone regeneration process in addition to their synergistic anti-cancer and anti-inflammatory enhancing effects. A more promising approach might be the combination with multitargeting agents such as the natural agent resveratrol, which mitigates the negative effects of estrogen deficiency at the epigenetic level by stimulating Runx2 and Osx, mainly through reducing the expression of pro-inflammatory cytokines [31].

## 4. Resveratrol

Resveratrol is a plant-derived bioactive polyphenol which belongs to the group of stilbenes (trans-3,5,4′-trihydroxystilbene) and was first isolated in 1939/40 from the roots of white hellebore (*Veratrum grandiflorum*) in Japan [142,143]. There are two known isoforms of resveratrol: *cis*-resveratrol, which is promoted by ultraviolet light and high pH, and *trans*-resveratrol, the more stable form, which is promoted by visible light, high temperature, and low pH [144].

High levels of this natural substance are produced within the skin of red grapes, which is considered as a major explanation for the “French paradox”, referring to the phenomenon of cardioprotection associated with the consumption of red wine in combination with a diet high in saturated fats in the French population [145,146]. In this context, evidence for the cardioprotective effects of resveratrol has been reported due to its ability to potentiate the anti-platelet effects of prostaglandins and inhibit low-density lipoprotein oxidation, both of which are known to be associated with the development of atherosclerosis [146]. Apart from its occurrence in the skin of grapes, there are multiple different plants and fruits, including cranberries, mulberries, blueberries, jackfruit, peanuts, eucalyptus, and Japanese knotweed [144], where the phytoalexin acts as a defense mechanism against bacteria, fungi, UV light, and other harmful factors [147].

**Figure 6:**
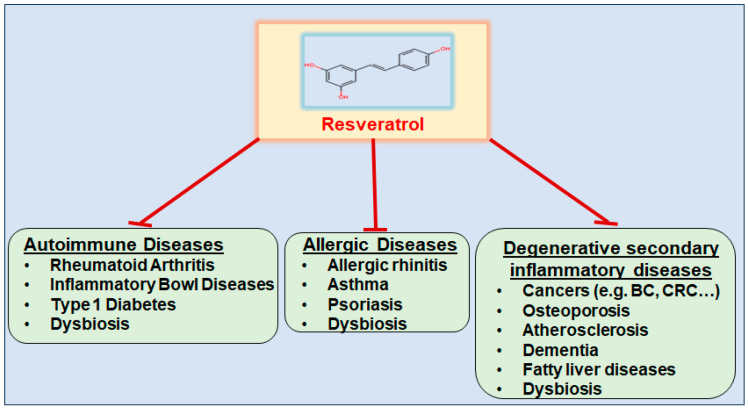
Poly-therapeutic effects of resveratrol with positive healing properties in inflammation and chronic diseases.

In recent decades, resveratrol has received significant medical attention for its anti-cancer, cardioprotective [146], anti-neurodegenerative [148], anti-osteoporosis [149], regenerative, and anti-aging properties [20]. Additionally, resveratrol has recently received attention as anti-adipogenic phytonutrient for the prevention and management of obesity and obesity-related metabolic syndrome [150], highlighting its potential role in the prevention of numerous chronic inflammatory diseases (Figure 6). The natural polyphenol is also a promising adjuvant approach to chemosensitization in the setting of conventional cancer therapies to prevent therapy resistance and reduce systemic side effects [151]. The great potential of the phytopharmaceutical is illustrated in particular by its property to act both catabolically, as on BC [56], and anabolically, as on OP [149]. As a bioactive molecule, resveratrol operates multifunctionally, especially with anti-inflammatory and immunomodulatory effects, since the polyphenol down-regulates NF-κB [30] and RANKL [16], thereby attenuating the gene expression of pro-inflammatory mediators [144]. Furthermore, resveratrol is known as a phytoestrogen through its capability to regulate the activity of α and β estrogen receptors [21], explaining its widely reported benefits in the cardiovascular system, central nervous system, and other tissues, including bone tissue. Phytoestrogens, such as resveratrol, are known as anti-oxidants and therefore significantly modify the cell microenvironment, especially that of tumor cells, thereby suppressing tumorigenesis [152].

Regarding pharmacokinetics, several data suggest that resveratrol has a high absorption but very low bioavailability when administered orally in humans [153]. While 75% of the oral dose is absorbed by transepithelial diffusion, its oral bioavailability is estimated to be less than 1% after intestinal and liver metabolism [153]. A majority of the data classify resveratrol as a safe and, in most cases, well-tolerated supplement, depending on the dosage [154]. In fact, the divergence in the functional dichotomy of resveratrol has been observed to affect cells in different ways, with cell survival being increased at low resveratrol concentrations and suppressed at high resveratrol concentrations [155,156].

### 4.1. Resveratrol as a Phytoestrogen and Epigenetic Modulator for Breast Cancer and Osteoporosis

**Figure 7:**
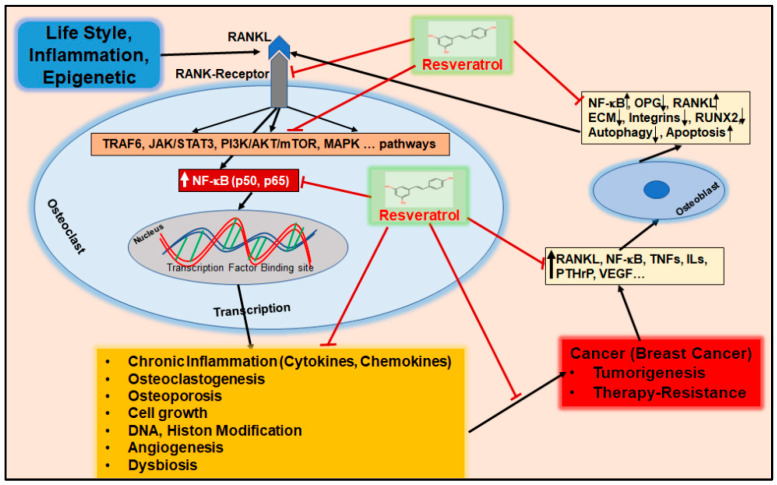
The association of resveratrol and signal transduction in chronic inflammation: breast cancer and osteoporosis. The up arrow (↑) indicates activation/increase/high regulation and the down arrow (↓) indicates decrease/decrease/regulation/suppression.

Everyday lifestyle factors characterize a systemic triangle of interactions between chronic inflammation, the development of BC cells, and damage to bone metabolism (Figure 7). Therefore, resveratrol’s numerous health-promoting regulatory mechanisms (Figure 6) are also being researched with regard to its influence on BC and OP.

#### 4.1.1. Resveratrol and Breast Cancer

Chronic inflammation triggered by an unhealthy lifestyle as well as environmental influences supports the acceleration of BC-promoting processes. Resveratrol is able to intervene in these complex mechanisms by means of broad-based signaling pathway modulation, alleviating the epigenetic-promoted, pro-inflammatory TME (Figure 7).

Numerous in vitro and in vivo studies have demonstrated the anti-proliferative, pro-apoptotic, anti-angiogenic, and chemosensitizing effects of resveratrol on various types of cancer, including BC [151], by attenuating pro-inflammatory mediators through the modulation of NF-κB and STAT3 signaling in BC cells (Figure 7) [157,158].

By positively regulating the TME, resveratrol has been shown to significantly affect tumorigenesis of BC by various mechanisms, including the induction of apoptosis via p53 signaling [159]. This finding was confirmed for other cancer cell lines such as colorectal cancer (CRC) cells, as evidenced by the reversal of p53 suppression by resveratrol [156]. In this context, a dose-dependent effect pattern of the natural polyphenol was recently confirmed through the inhibition of the NAD-dependent protein deacetylase Sirt-1 and the acetylation of p53 protein at higher resveratrol concentrations [156]. Another mechanism by which resveratrol has been shown to stimulate the apoptosis of triple-negative BC cells is by modulating the expression of DNA polymerase delta 1, supporting its multitarget effect at the epigenetic level [160]. Moreover, resveratrol analogs have been shown to induce apoptosis through the mitochondrial pathway, including the activation of caspase-3 and poly (ADP-ribose) polymerase (PARP) [161], as well as to increase senescence via signaling pathways such as p53/p21, leading to tumor growth arrest in BC cells [162]. In addition, the use of resveratrol in different cancer models in animals has had different effects, namely positive, negative, or neutral. These have depended on the respective dosage, tumor model, sex and strain of the animals, and method or time of resveratrol application [23,156,163].

Resveratrol has further been demonstrated to enhance the effectiveness of chemotherapy in BC by synergistically attenuating cancer cell plasticity through the modulation of PI3K/Akt, Smad, NF-κB, JNK, and ERK in BC cells (Figure 7) [158]. The chemosensitizing effect of resveratrol and other polyphenols, including curcumin [164] and calebin A [165], has been confirmed in other cell lines, such as CRC, by mechanisms such as decreasing β1-integrin expression, associated with reduced invasion and cancer cell plasticity [166]. Another synergistic role of resveratrol in chemotherapy is based on its property as an autophagy inducer, which triggers excessive autophagy in cancer cells, impairing the protective function of autophagy in cancer cells and promoting cell processes such as apoptosis [80]. In this context, a reduction in protective autophagy flux by resveratrol was reported in association with impaired repair of double-strand breaks in BC cells, synergistically enhancing the effect of the PARP inhibitor talazoparib through dual inhibition of the PI3K/Akt signaling pathway [167]. As discussed in Section 3.1.1., deficiency in or impairment of autophagy in BC is often associated with low expression of autophagy genes including Beclin-1 [79]. In this regard, resveratrol has been shown to increase the expression of appropriate Beclin-1 and LC3β genes in BC cells, associated with the formation of autophagic vacuoles, resulting in proliferation suppression via the Sirt-3/Adenosine monophosphate-activated protein kinase (AMPK) axis [168]. In addition, other study data provide evidence that the phytopharmaceutical stimulates autophagy in cancer cells through Beclin-1-independent pathways, including the modulation of Wnt/β-catenin signaling and direct inhibition of the mTOR-Unc-51-like kinase 1 pathway via ATP competition, indicating its multitarget mode of action and the importance of autophagy [56,169] (Figure 7). Additionally, the mechanism of enhanced exocytosis was shown to be associated with resistance to therapy in conjunction with up-regulated NF-κB signaling, leading to the overexpression of chemoresistance proteins such as multidrug resistance protein (MDR1) and multidrug resistance-linked protein 1 (MRP1) in BC [170]. In this regard, resveratrol has been demonstrated to act as a chemosensitizer by modulating the expression of MDR1 and MRP1 in multidrug-resistant BC cells [171]. This is in accordance with other data demonstrating the reversible effect of resveratrol on multidrug resistance in cancer cells by suppressing mTOR and nuclear factor erythroid 2-related factor 2 activation through the acceleration of p62 degradation (Figure 7) [172].

Resveratrol analogs further suppress epigenetic-triggered hypoxia-inducible factor 1-alpha (HIF-1α) and VEGF in BC, thereby sensitizing the effect of radiotherapy effectively through the modulation of the hypoxic state and blood flow under hypoxia conditions, leading to the inhibition of angiogenesis [173]. Moreover, resveratrol has recently been reported to repair radiation-induced DNA damage in skin tissue via a novel AMPK/Sirt-7/high-mobility-group-box-1 (HMGB1) regulatory axis, underscoring the multifunctional mode of action of resveratrol [174].

An important ability of resveratrol is its immunomodulatory function, which has also been reported in association with greater efficacy of radiotherapy and has been positively correlated with a reduction in tumor-derived T_reg_ cells, resulting in increased anti-tumor immunity [175]. Further in vivo studies support the immunomodulatory effect of resveratrol analogs on tumor-derived T_reg_ cells, resulting in enhanced anti-tumor immunity through increased tumor-specific cytotoxic T-lymphocyte responses and CD4^+^ T-cells, supporting the use of resveratrol analogs as adjuvant agents for BC immunotherapy [176].

More interestingly, resveratrol is able to synergistically enhance the effect of aromatase inhibitors on hormone-sensitive BC cells by modulating aromatase activity at the epigenetic level, sustaining the hypothesis of its co-therapeutic benefits [177]. Further, resveratrol also appears to be a promising natural substance for the prevention of BC recurrence during pregnancy, without negative effects on the embryo [178]. Indeed, positive effects of resveratrol on embryogenesis and the prevention of BC during pregnancy and pregnancy complications as well as on improvements in reproductive health have been reported [179,180].

#### 4.1.2. Resveratrol and Osteoporosis

A wide range of epigenetic-influenced physical and psychosocial symptoms can occur in BC patients [181], making the early prevention of secondary diseases, such as secondary OP, essential and requiring an appropriate integrative therapeutic approach [135]. As outlined in Section 3.2, conventional BC treatment often leads to estrogen deficiency and exacerbates chronic inflammation, known to accelerate bone resorption and decrease bone formation [60,182]. In this regard, the pro-inflammatory osteoporotic microenvironment is a major driver of epigenetic alterations, leading to significant dysregulations of bone remodeling through enhanced osteoclastogenesis and attenuated osteogenesis [183].

Resveratrol has been shown to influence bone resorption through multitarget effects by modulating the osteoporotic microenvironment through regulating key pathways including NF-κB and RANKL [16] (Figure 7). In vivo evidence from a postmenopausal OP rat model has demonstrated reduced levels of pro-inflammatory cytokines associated with a small increase in bone mineral density (BMD) after only two weeks of resveratrol supplementation [184]. The study further suggested a restoration of the RANKL/OPG ratio [184], which is consistent with findings showing that resveratrol suppresses RANKL-induced NF-κB activation along with suppressing IκBα kinase and IκBα phosphorylation and degradation [16]. In addition, an up-regulation of Sirt-1 by resveratrol has been demonstrated, reversing NF-κB acetylation through RANKL-up-regulated histone deacetylase p300 in a time- and concentration-dependent manner [16], which is in accordance with evidence that used a postmenopausal rat model [185]. Sirt-1 activation is known to be one of the key molecules in bone homeostasis [18,47] and is established as one of the major subcellular targets of the epigenetic modulator resveratrol [31].

Further results have demonstrated that resveratrol reverses the suppression of MSC differentiation to osteoblasts by modulating NF-κB signaling and pro-inflammatory cytokines through the up-regulation of Sirt-1 and Runx2 [30]. Additionally, studies revealed that the combination of resveratrol with other polyphenols, such as curcumin, synergistically modulates the NF-κB pathway in chondrocytes, evidenced by a reduction of inflammatory mediators [186]. Resveratrol and curcumin have further shown a synergetic anti-apoptotic effect on chondrocytes through decreased expression of B-cell lymphoma (Bcl)-2, Bcl-xL, and TNF-α receptor-associated factor 1 [186]. Moreover, resveratrol prevents osteoblasts from undergoing apoptosis through modulating pro-inflammatory cytokines and caspase-3 expression, which is consistent with results demonstrating that resveratrol suppresses IL-1β-induced stimulation of caspase-3 expression and cleavage of PARP in chondrocytes, associated with an increase in the expression of ECM molecules, including collagen type II and β1-integrin [187]. It is also interesting that resveratrol increases ECM molecules, including collagen type I and osteocalcin, in high-density bone cultures [16]. In addition, the phytoestrogen promotes osteogenesis in MSC and preosteoblastic cells by stimulating the expression of Runx2 via the up-regulation of Sirt-1 and suppression of PPAR-γ activity in vitro [31]. Recent in vivo observations support these findings by showing increased expression of osteogenic markers such as Runx2, OPG, and OCN upon resveratrol supplementation [188]. Other research data have provided in vivo evidence for increased expression of bone morphogenic protein (BMP)-2, BMP-7, and OPN in rats, highlighting resveratrol’s effect on bone repair [189].

A beneficial characteristic of resveratrol consists in its modulation of processes such as autophagy and apoptosis in osteoblasts and MSC under osteoporotic microenvironments in in vitro and in vivo OP models, promoting cell survival, function, and differentiation, which is crucial for the regeneration of ECM in bone tissue [190] (Figure 7). Moreover, results from a rat model with postmenopausal OP have demonstrated that resveratrol increases autophagy, thereby promoting osteoblast differentiation [191]. These findings are in accordance with observations suggesting that the number of mouse pre-osteoblasts rises under resveratrol treatment through increased autophagy [190], which can be interpreted as protection against epigenetic changes. Furthermore, in vivo results demonstrated a protective role of the natural polyphenol on osteoblasts by increasing the expression of Beclin-1 and LC3 through up-regulation of Sirt-1, associated with suppression of the mTOR/Akt/PI3K pathway [192]. Considering that the underlying rat model relied on dexamethasone-induced OP, resveratrol can be considered a beneficial adjunctive agent for symptomatic treatment using glucocorticoids, known to promote bone resorption processes [193]. Regarding osteocytes, the grape-derived compound has also been shown to protect osteocytes against oxidative stress through AMPK/JNK1 activation, thereby inducing autophagy and suppressing apoptosis [11]. There is evidence that resveratrol increases mitochondrial biogenesis in MSCs, associated with enhanced osteogenic differentiation, which supports the assumption that inducing autophagy in bone cells may also promote energy homeostasis [194]. By reducing epigenetic-associated oxidative stress through the AMPK pathway, resveratrol has been reported to modulate the expression of senescence-related genes, including p16, p21, and p53, resulting in enhanced osteogenic differentiation [195]. In regard to p53, resveratrol has been demonstrated to partially reverse the p53-induced inhibition of osteogenic differentiation by modulation of mouse double minute 2 homolog (MDM2)-mediated p53 degradation [149].

Furthermore, the function of this natural substance as a phytoestrogen confirms its significance for bone anabolism, allowing resveratrol to activate estrogen receptor-α, which has been linked to increased osteogenesis [196]. Moreover, the aforementioned up-regulation of Sirt-1 by resveratrol in association with increased autophagy and decreased apoptosis in osteoblasts can be considered as evidence for the phytoestrogenic effect of resveratrol, since estrogen analogues have recently been shown to have a comparable effect [45,47,197]. Indeed, findings from ovariectomized rats indicate no adverse side effects from long-term daily use of resveratrol in organs with high estrogen sensitivity, such as the uterus [198]. These findings are consistent with a recent meta-analysis providing evidence for an increase in BMD in OP rat models through improvement in bone microstructure and regulation of calcium and phosphorus metabolism by resveratrol [199].

Another argument for the beneficial multitarget effect of the phytopharmaceutical on bone formation consists in its indirect effect on potentiating vitamin D nuclear signaling, associated with enhanced 1,25-Dihydroxyvitamin D3 binding to vitamin D3 receptors (VDRs), activation of the retinoid X receptor, and stimulation of Sirt-1 [200]. These effects of resveratrol may also be beneficial in BC, as a meta-analysis has found low levels of vitamin D in many cases of newly diagnosed BC patients [201], which is supporting evidence that vitamin D controls tumor growth and CD8^+^ T-cell infiltration in BC [202].

### 4.2. Clinical Trials with Resveratrol

In view of the currently inadequate conventional clinical treatment options for patients with BC-related OP, the following subsections present clinical evidence for the positive effects of resveratrol on the prevention and adjuvant treatment of BC and OP. The particular importance of this phytonutrient lies in its function as a polyactive catabolic and anabolic phytopharmaceutical, which plays a decisive role in BC-related OP.

#### 4.2.1. Breast Cancer

There is a growing clinical awareness of the importance of an anti-inflammatory lifestyle in the prevention and treatment of BC, a routine that includes adequate exercise, mental stability, and an anti-inflammatory diet [63,64]. In clinical practice, it has been shown that anti-inflammatory dietary habits such as a high daily intake of polyphenols (median: 2230 mg/day) contribute to a reduction in inflammatory markers, including plasma C-reactive protein (CRP) in BC patients [203], suggesting a chemopreventive effect, as elevated CRP levels have been shown to correlate positively with the development of BC [204]. More specifically, resveratrol supplementation has also been demonstrated to reduce levels of pro-inflammatory cytokines and VEGF in patients with high risk of BC, supporting the anti-inflammatory effects of polyphenols [205,206] (Table 3). Several meta-analyses of more than 15 randomized controlled trials, each with more than N = 600 subjects, confirmed the anti-inflammatory effects of resveratrol, primarily reporting a reduction in TNF and high-sensitivity CRP levels [207,208]. Indeed, there is clinical evidence that obese and young individuals particularly profit from the anti-inflammatory effect of resveratrol demonstrated by reduced TNF-α levels [208]. As a potent epigenetic modulator, serum levels of the phytopharmaceutical have been shown to correlate directly with reverse methylation of tumor suppressor genes such as Ras-associated domain family (RASSF)-1, which has been associated with reduced PGE2 levels in women predisposed to BC (Table 3) [205].

Besides chemoprevention, the anti-inflammatory effects of resveratrol are fundamental in reducing the adverse effects of chemotherapy and radiotherapy while synergistically enhancing BC therapy effectiveness [209,210] (Table 3). In this context, an interesting study on organoid models of advanced BC showed that resveratrol was more effective in terms of cell death rate and had a broader spectrum against different subtypes of advanced hormone-sensitive BC compared to conventional BC drugs such as fulvestrant, paclitaxel, and gemcitabine [211]. These results underline the versatile effect of resveratrol as a chemosensitizer in BC, both by sensitizing BC cells and by the synergistic effect of resveratrol itself with chemotherapeutic agents.

With regard to side effects due to radiation in BC patients, reduced skin toxicity has been reported in BC patients receiving adjunctive therapy with 50 mg resveratrol daily in combination with vitamin C, antocyanins, and lycopene 10 days before and 10 days after radiotherapy [210]. This clinical evidence is consistent with in vivo data demonstrating the radioprotective effect of resveratrol on healthy tissue, which should be investigated in further clinical trials [212]. The radioprotective effect of the natural polyphenol has been shown by promoting skin regeneration through NF-κB modulation and stimulating collagen synthesis by acting anabolically as a phytoestrogen through the activation of estrogen receptors in keratinocytes [213]. In addition, resveratrol is hypothesized to activate VEGF to stimulate the regeneration of damaged skin tissue while enhancing the effect of radiotherapy on cancer cells (Table 3), highlighting its regenerative and catabolic effects depending upon tissue type and the cell microenvironment [173,210].

Interestingly, there is increasing discussion of another mechanism which has been demonstrated to synergistically enhance the effect of BC therapies, a process known as calorie restriction [214]. Notably, resveratrol is recognized to effectively mimic calorie restriction via multiple pathways, including the modulation of IGF1 [215]. A crucial signaling pathway associated with calorie reduction is the inhibition of mTOR/PI3K/Akt, which has been described in the previous section in connection with autophagy and resveratrol [167]. Clinically, monoclonal antibodies against mTOR, such as rapamycin, are used in BC to reduce chemoresistance but have been demonstrated to have various negative systemic side effects which may be explained by their inhibitory effect on mTOR autophagy in physiological tissues, thereby attenuating cell vitality [216]. Therefore, multifunctional agents such as resveratrol represent a promising approach to modulate mTOR signaling depending on the cell microenvironment, acting both as a positive trigger of autophagy synergistic to chemotherapy and inducing apoptosis through excessive autophagy in tumor cells, as discussed in Section 4.1.1, without negative side effects [167]. This underlines that adjuvant therapy crucially needs to have the properties of reducing side effects in physiological tissue while synergistically enhancing the conventional form of therapy.

Additionally, there is evidence that a high-polyphenol diet may improve BC prognosis by modulating neutrophil/lymphocyte and lower platelet/lymphocyte ratios [203], as both have been reported as clinical indicators of a poor BC prognosis [217,218]. In this regard, resveratrol is recognized for modulating platelet metabolism and function by reducing the activity of enzymes involved in glycolysis and oxidative metabolism in platelets, supporting a beneficial effect of polyphenols on the platelet/lymphocyte ratio [219]. This suggests a multifunctional way to inhibit thromboxane biosynthesis in BC patients, in contrast to synthetic drugs such as aspirin [220], which may cause additional challenges such as drug resistance.

In terms of long-term follow-up of BC patients, a recently published study of clinical interest highlighted a significant challenge by showing that IL-17 remains significantly elevated in patients with early-stage BC even after adjuvant chemotherapy and endocrine therapy, suggesting that new solutions are needed [221]. Of relevance in this context, resveratrol has been shown in preclinical studies to modulate Th17 differentiation via the activation of Sirt-1, leading to the suppression of the p300-activated STAT3 signaling cascade, thereby stimulating T_reg_ and Th2 differentiation [222], suggesting that this natural multifunctional agent may offer a promising approach to restore enhanced immune function in BC patients.

Another relevant clinical finding supporting the beneficial effects of resveratrol in BC suggests that the polyphenol positively affects estrogen metabolism by modulating levels of sex steroid hormone-binding globulin (SHGB), contributing to a significant decrease in bioavailable estrogen in women with high BMI [223] (Table 3). Evidence for a beneficial effect of increased SHGB in BC is also based on findings showing that SHGB reverses the anti-apoptotic effect of estradiol in BC cells, with a binding site on BC cells that has been shown to inhibit estradiol-induced cell proliferation after SHGB binding [224]. Indeed, other clinical studies have confirmed the modulatory effect of resveratrol on SHGB expression [225].

Clinical evidence for the impact of lifestyle changes, including an anti-inflammatory diet with regular consumption of polyphenols, on the prevention and adjuvant treatment of BC was reported by a study of a premenopausal woman with estrogen-sensitive stage IV BC with spontaneous remission 6 months after the initiation of evidence-based phytonutrient therapy [226]. The supplementation of individual polyphenols in the BC patient followed a comprehensive adjustment of her diet, replacing the patient’s original high-calorie diet, mainly based on simple carbohydrates, with one containing more fiber, fresh fruits, and vegetables [226]. Instead of packaged fruit juice and sweetened drinks, the patient switched her consumption to beverages with natural polyphenols, such as green tea and Malaysian cocoa [226]. After 6 months, serum estradiol levels were reduced by more than 50% of the original level, supporting the hypothesis that phytoestrogens including resveratrol have a beneficial regulating effect on BC due to their multifunctional actions [226]. The same research group also reported a beneficial effect of the same concept of phytonutrient therapy on a patient with CRC [227] and a patient with lung cancer [228]. These individual case studies underscore the importance of lifestyle changes, such as an adequate anti-inflammatory diet including phytochemicals, in cancer treatment regimens and further support the postulated beneficial co-therapeutic effects of resveratrol supplementation in conjunction with conventional BC therapy based on the fact that epigenetic changes in BC are reversible (Table 3).

**Table 3:** Clinical trials with resveratrol in breast cancer prevention and adjunct therapy.

Study Participants	Year of Publication	Study Type	Resveratrol Treatment	Clinical Impact	Reference
N = 36 healthy premenopausal women (36 ± 8 years)	2011	Crossover design	Oral: 8 ounces (237 mL) red wine daily for 21 days	Lower SHBG levels; higher free testosterone and LH levels. Suggestive of hypothalamic up-regulation in response to lower estrogen levels. Postulated nutritional aromatase inhibitor and no increased risk factor for BC development.	[229]
N = 39 women; high BC risk; 57.5 ± 3.5 years	2012	Prospective, double-blind, and placebo-controlled	Oral: 5 or 50 mg twice/day vs. placebo for 3 months	Suppression of BC-promoting prostaglandin and DNA methylation. Proposal of BC risk reduction.	[205]
N = 71 (N = 30 Ixor group) BC patients Age between 30–80 years	2012	Prospective, randomized, placebo-controlled observational study	Oral: 25 mg trans-resveratrol twice/day combined with lycopene, vitamin C, and anthocyanins 10 days before radiation to 10 days after the treatment	Reduced skin toxicity due to external beam radiation therapy compared to control group.	[210]
N = 34 women; high BC risk; 58 ± 8 years	2014	Randomized controlled clinical trial	Oral: 1000 mg/day for 3 months	Hormone balance supported by estrogen regulation. Assumed reduction of BC risk.	[223]
N = 300 (Subgroup of N = 100, N = 49 Resveratrol Ixor Group) BC patients, median age 56 years (range 28–80 years)	2014	Prospective, randomized, placebo-controlled observational study	Oral: 25 mg trans-resveratrol twice/day combined with lycopene, vitamin C, and anthocyanins from 10 days before radiation treatment to 10 days after the treatment	Reduced skin toxicity in breasts with a volume lower than 500 mL and in those who receive a radiation dose between 107% and 110% of the prescribed dose. Chemoprotective effect in patients undergoing chemotherapy with anthracyclines/taxanes.	[209]
N = 1; premenopausal woman with stage IV BC, 48 years old	2015	Single case study	Oral: 400 mg trans-resveratrol thrice a day combined with broad-based phytonutrient therapy	“Spontaneous regression”. Decreased levels of BC serum marker (CA15-3), pro-inflammatory markers (IL-6, hs- CRP, IL-6), estradiol, and cortisol. Decreased BMI.	[226]
N = 27 women and 1 man; BC patients; >18 years	2019	Randomized controlled clinical trial	Oral: 473.7 mg phenolics (containing 53.85 mg resveratrol) thrice/day vs. placebo, 6 ± 2 days	Detection in metabolic end products as well as healthy and malignant tissue. Possible consideration of a long-term chemopreventive effect.	[230]
39 women; BC patients; 54 ± 11 years	2021	Randomized controlled clinical trial	Oral: 296.4 mg phenolics (containing 65 mg resveratrol) thrice/day vs. placebo for 5 ± 2 days thrice/day vs. placebo for 5–7 days	Confirmation of detection in metabolic end products. Final recommendation of polyphenol co-therapy for BC remained open.	[231]

Abbreviation: BC—breast cancer, DNA—deoxyribonucleic acid, SHBG—sex hormone-binding globulin, LH—luteinizing hormone, CA—cancer antigen, IL—interleukin, hs-CRP—high-sensitivity C-reactive protein, BMI—body mass index.

#### 4.2.2. Osteoporosis

The importance of phytochemicals in the clinical prevention of OP (Table 4) has been increasingly evidenced by the modulation of osteogenic biomarkers, leading to a reduction in fracture risk through an increase in BMD, when other parameters such as an adequate supply of vitamin D and calcium and sufficient physical activity are ensured [54]. Specifically, a screening study with over N = 3000 perimenopausal women has demonstrated that specific dietary habits including bioactive polyphenols positively correlate with beneficial effects on BMD at the femoral neck and lumbar spine associated with reduced levels of bone resorption markers [232]. Similar findings have been confirmed for several subgroups of polyphenols, such as flavonoids and stilbenes, including resveratrol [233]. Regarding resveratrol, recent clinical evidence has shown that a daily dietary intake of resveratrol correlates with a lower risk of hip fracture, particularly in women and less obese participants [234] (Table 4).

Furthermore, 1 year of supplementation with resveratrol in combination with other phytoestrogens such as equol and fermented soy was associated with an increase in total body BMD and an improvement in bone turnover parameters such as deoxypyridinoline (DPD), osteocalcin, and bone-specific alkaline phosphatase (BAP) compared to placebo [235] (Table 4).

This finding is consistent with the results of the longest clinical study to date, the 2-year *Resveratrol for Healthy Aging in Women* (RESHAW) study, which reported increases in BMD at the lumbar spine and femoral neck after 1 year of supplementation in healthy postmenopausal women [54]. The enhancement of BMD in the femoral neck under resveratrol supplementation has been found to correlate inversely with a decrease in c-terminal telopeptide type 1 [54], a bone resorption marker, reported to be increased in postmenopausal women with OP [103]. Recently, additional results from the *RESHAW* study (Table 4) have shown that increased lumbar BMD corresponds with an increase in bone turnover markers, including ALP, along with the modulation of the paracrine growth factor CNP [236]. Of clinical significance, supplementation with resveratrol has been shown to improve femoral neck T-scores along with a reduced 10-year probability of major hip fractures [54], which is of great clinical importance, as the femoral neck is one of the most common fractures in OP patients [60]. Indeed, femoral neck Sirt-1 expression has been shown to be reduced in OP patients [237], supporting the bone-protective influence of resveratrol, based on the fact that the polyphenol targets Sirt-1 as its main intracellular molecule [16,31]. As an activator of Sirt-1 in the osteoporotic microenvironment, resveratrol is recognized to stimulate Runx2 and Osx, consequently making this multifunctional molecule a promising next-generation agent for OP therapy, including the aspect of supporting necessary bone healing after OP fractures [16,18,30].

In addition to postmenopausal women, resveratrol has also been shown to maintain or increase BMD and bone mineralization in individuals at risk for secondary OP, including those with high alcohol consumption, obesity, and primary chronic inflammatory diseases such as type 2 diabetes mellitus [54,238,239]. In this context, resveratrol supplementation has been shown to be associated with increased bone markers, including ALP and BAP [238,239], supporting in vitro and in vivo evidence that resveratrol modulates RANKL and stimulates Runx2 and OC [30,31]. Specifically, a positive clinical association between elevated serum BAP and increased BMD at the lumbar spine has been demonstrated in a dose-dependent manner [240]. These results suggest that resveratrol supplementation could have a similar positive effect in patients with secondary OP induced by conventional BC treatment.

Another important benefit of using resveratrol in OP patients is its analgesic effect, which has been suggested as an adjunct to conventional pain management [16,18,30]. In fact, both short-term (3.5 months) and long-term (24 months) supplementation with this multifunctional compound has been shown to correlate with a reduction in musculoskeletal pain in postmenopausal women [241,242] (Table 4). This is achieved through resveratrol’s established modulation of NF-κB at the COX/PGE axis, which is a major clinical advantage over monotarget analgesics such as NSAIDs [243]. Importantly, no serious side effects have been demonstrated in postmenopausal women taking the agent over a two-year period, but rather a positive increase in general well-being and an improvement in postmenopausal symptoms [242].

Finally, it is important to consider that physical activity is an important supportive measure during treatment with polyphenols such as resveratrol, as osteocytes are mechanosensitive cells that require sufficient physical activity to maintain balanced bone remodeling [37]. In this context, green tea polyphenols in combination with tai chi have been reported to increase BAP, improve the ratio of BAP to tartrate-resistant acid phosphatase (TRAP), and significantly improve muscle strength in a 6-month trial group of N = 171 postmenopausal women with osteopenia [244]. Furthermore, resveratrol supplementation combined with walking and full-body resistance training twice a week for a total of 4 months has been associated with improved epigenetic conditions such as skeletal muscle mitochondrial function and mobility-related indices of physical function [245], encouraging further clinical trials with resveratrol in combination with regular physical activity for patients with BC-related OP [54].

**Table 4:** Clinical trials with resveratrol in osteoporosis prevention and adjunct therapy.

Study Participants	Year of Publication	Study Type	Resveratrol Treatment	Clinical Impact	Reference
N = 74 obese men with metabolic syndrome; 49.3 ± 6.3 years	2014	Randomized, double-blind, placebo-controlled trial	Oral: 150 mg or 1000 mg resveratrol vs. placebo for 4 months	Increase in BAP. Promotion of bone formation as well as mineralization.	[240]
N = 24 obese (BMI: 34 ± 0.7) non-diabetic men; resveratrol group: N = 12; 44.7 ± 3.5 years	2014	Randomized, double-blind, placebo-controlled, parallel-group design	500 mg resveratrol thrice a day for 4 weeks	Increased plasma levels of BAP.	[239]
N = 80 healthy postmenopausal women; 61.5 ± 0.9 years; 11.6 ± 1.0 years postmenopausal, average BMI: 26.7 ± 0.6 kg/mL normotensive	2017	Randomized, double-blind, placebo-controlled, two period crossover intervention trial	Oral: 75 mg trans-resveratrol twice daily for 14 weeks	Reduced pain experience. Improved general well-being.	[241]
N = 192 patients with type 2 diabetes Age ± 40 years, BMI < 35 kg/m^2^	2018	Double-blind randomized controlled trial	Oral: 500 mg or 40 mg resveratrol for 6 months	Increase in osteogenic markers: BAP in both groups. Whole-body BMD remained significantly higher with resveratrol compared to placebo; increased vitamin D.	[238]
N = 146 healthy postmenopausal women; 64.3 ± 1.3 years	2020	Randomized, placebo-controlled trial	Oral: 75 mg resveratrol twice/day vs. placebo for 24 months	Improvement in bone perfusion and BMD. Reduction in fracture risk.	[54]
N = 125 healthy postmenopausal women	2021	Randomized, double-blind, placebo-controlled, two period crossover trial	Oral: 75 mg resveratrol twice a day for 24 months	Improved pain perception, especially in overweight individuals. Improved somatic postmenopausal symptoms and general well-being.	[242]
N = 1.065 patients with hip fracture incident; 70.7 ± 7.3 years	2023	1:1 age- (±3 years) and gender-matched case–control study	Average total resveratrol intake: 14.1 ± 54.6 μg/day; major food sources included grapes, apples, and nuts	Lowered risk of hip fracture was positively correlated with greater intake of dietary resveratrol and resveratrol-rich foods.	[234]
N = 60 healthy postmenopausal women; 52.09 ± 1.71 years	2023	Randomized, placebo-controlled trial	Oral: 200 mg fermented soy with 25 mg resveratrol and 10 mg equol vs. placebo for 12 months	Positive modulation of bone mineral density and bone turnover parameters.	[235]
N = 125 postmenopausal women with mild osteopenia, Age 45–85	2023	Randomized controlled clinical trial	Two-year period of study. Year one: placebo or oral resveratrol 75 mg twice daily; year two: switched to placebo or resveratrol, respectively	Suppression of C-type natriuretic peptide associated with increased vertebral bone density. Increased ALP. Inverse association of NTproCNP and positive association of OC with BMD at the lumbar spine.	[236]

Abbreviation: BMI—body mass index, BAP—bone alkaline phosphatase, BMD—bone mineral density, ALP—alkaline phosphatase, NTproCNP—N-Terminal pro natriuretic peptide, OC—osteocalcin.

To the authors’ knowledge, there are no clinical studies to date on preventive and adjuvant therapy with resveratrol for secondary OP induced by BC therapy. However, current in vivo and in vitro results suggest that resveratrol provides protective and adjuvant therapeutic benefits in BC without adverse effects on estrogen-sensitive tissues (Table 5). In particular, combined grape polyphenols in combination with trace elements such as zinc have been associated with a delay in BC-induced bone loss and a catabolic effect on BC tumorigenesis [246,247] (Table 5).

## 5. Summary and Perspective

In this review, the use of resveratrol in patients with secondary OP induced by BC treatment is considered promising for maintaining long-term bone health by stimulating bone regeneration while synergistically supporting catabolic effects on BC tissue. It is important to emphasize that we propose the supplementation of the natural polyphenol as an adjuvant factor in the context of a holistic, personalized therapeutic concept that, in addition to an anti-inflammatory diet, also includes regular physical exercise and the prevention of psychosocial stress as well as the avoidance of noxious substances such as nicotine and alcohol. Considering molecular evidence of lifelong reversible epigenetic changes, a long-term adapted lifestyle is of crucial significance, which also applies to the daily supplementation of natural active compounds. In this context, resveratrol is suggested as a promising prophylactic and therapeutic supplement to inhibit tumorigenesis as well as therapy resistance and undesirable side effects in BC patients while promoting tissue regeneration, including bone tissue.

Further research on the effects of resveratrol on bone metabolism/health in BC patients is crucial. In particular, longitudinal, randomized, placebo-controlled clinical trials are needed to better understand the exact dosage, duration of treatment, drug interactions, and potential side effects of resveratrol in BC patients. However, the natural multifunctional agent is considered beneficial in moderate doses mainly because of its flexible, poly-targeted mode of action, which is oriented towards the cellular microenvironment and is considered one of its greatest advantages over monotargeted drugs such as bisphosphonates, making the natural compound a safe supplementary option for many BC patients, especially for those who may already be managing complex medical conditions and medications.

Interdisciplinary collaborations between oncologists, endocrinologists, trauma surgeons, orthopedists, radiologists, nutritionists, and basic researchers is needed to develop a prophylactic, holistic, personalized therapeutic concept that enables effective strategies to maintain long-term bone health in BC patients, leading to increased quality of life.

## Figures and Tables

**Table 5:** Resveratrol’s therapeutic possibilities in breast cancer-associated osteoporosis.

Study Concept	Year of Publication	Resveratrol Treatment	Resveratrol’s Impact	Reference
In vivo, OVX rats	2005	45 mg/kg for 90 days	Reduction in endocortical bone absorption alongside increased bone formation and mineral density. Proposal of preventive potential against postmenopausal OP without adverse effects on estrogen-sensitive tissues such as the endometrium.	[248]
In vitro, MG-63, MC3T3-E1 cells In vivo, OVX Wistar rats and BC SCID mice	2007	1–10 µM for 7 days 10 mg/kg/every 2 day for 10 weeks	Increase in osteogenic response and osteoblast differentiation. Prevention of bone loss and BC progression. Conclusion of high effectiveness in postmenopausal OP without forced BC risk.	[249]
In vitro, MDA-MB-231 cells In vivo, nude mice	2009	0.5–20 µM for 4 days 5 mg/kg/3 times a week for 77 days	Inhibition of BC cell proliferation and migration. Significant reduction in NF-κB-related inflammation, tumor size, and bone metastasis. Greatest effect with combined grape polyphenols.	[247]
In vitro, primary human BC cells	2013	5–100 µM for 24 h	Deceleration of BC cell proliferation and induction of apoptosis by inhibiting bone marrow stromal-cell antigen (BST2).	[250]
In vivo, OVX Wistar rats	2014	20, 40, or 80 mg/kg for 12 weeks; initiated at week 2 after OVX	Improved BMD and trabecular microarchitecture without adverse effects on estrogen-sensitive tissues such as the endometrium. Bone-protective effect with 80 mg/kg resveratrol almost equivalent to control group with estradiol replacement.	[198]
In vivo, BC Sprague-Dawley rats	2015	0.2 mg/kg/d for 40 days plus zinc	Supported delay in or prevention of BC-associated bone loss.	[246]
In vivo, OVX Sprague Dawley rats	2020	10–40 mg/kg/d for 8 weeks	Known reduction in BC risk. Suppression of osteoclasts and simultaneous promotion of osteoblasts despite postmenopausal OP.	[191]

Abbreviations: BC—breast cancer, NF-κB—nuclear factor kappa-light-chain-enhancer of activated B cells, SCID—severe combined immunodeficiency, OVX—ovariectomized, BMD—bone mineral density.

## Data Availability

Original results are included in the present article. Additional inquiries can be directed to the corresponding author.

## Abbreviations

Akt	protein kinase B
ALP	alkaline phosphatase
AMPK	adenosine monophosphate-activated protein kinase
BAP	bone-specific alkaline phosphatase
BC	breast cancer
BMP	bone morphogenic protein
BMD	bone mineral density
COX	cyclooxygenase
CNP	C-type natriuretic peptide
CRC	colorectal cancer
CRP	C reactive protein
CXCL	CXC motif ligand
DKK	Dickkopf-related protein
ECM	extracellular matrix
EMT	epithelial–mesenchymal transition
ERK	extracellular signal-regulated kinase
Fas	apoptosis antigen 1
FGF	fibroblast growth factor
HER	human epidermal growth factor receptor
HGF	hepatocyte growth factor
HIF-1α	hypoxia-inducible factor 1-alpha
ICAM-1	intercellular adhesion molecule 1
IFN	interferon
IGF	insulin-like growth factor
IkBα	inhibitor nuclear factor of kappa B α
IKKα or IKKβ	inhibitor of kappa B kinase
IL	interleukin
IRF	interferon regulatory factor
JAK	Janus kinase
JNK	c-Jun *N*-terminal kinase
Ki67	Kiel antigen 67
KPNA2	Karyopherin α-2
LC3	microtubule-associated protein 1 light chain 3
MAPK	mitogen-activated protein kinase
M-CSF	macrophage colony-stimulating factor
MDM2	mouse double minute 2 homolog
MDR1	multidrug resistance protein 1
MEK	mitogen-activated protein kinase kinase
MMP	matrix metalloproteinase
MRP	multidrug resistance-linked protein
MSC	mesenchymal stem cell
mTOR	mammalian target of rapamycin
NF-κB	nuclear factor kappa-light-chain-enhancer of activated B cells
NFAT	nuclear factor of activated T-cells
NSAID	non-steroidal anti-inflammatory drug
OCN	osteocalcin
OP	osteoporosis
OPG	osteoprotegerin
Osx	Osterix
OVX	ovariectomized
PARP	poly (ADP-ribose) polymerase
P2Y	purinoceptor 2
PCNA	proliferating cell nuclear antigen
PDGF	platelet-derived growth factor
PI3K	phosphatidylinositol 3-kinase
PTHrP	parathyroid hormone-related protein
RANK	receptor activator of nuclear factor (NF)-kB
RANKL	receptor activator of nuclear factor (NF)-kB ligand (RANKL)
Ras	rat sarcoma
RASSF	Ras-associated domain family
Runx2	Runt-related transcription factor 2
Sirt-1	silent information regulator sirtuin 1
sgp130	soluble glycoprotein 130
SHP2	Src homology-2 domain-containing protein tyrosine phosphatase-2
SOX9	SRY-related homeobox gene 9
SHGB	sex steroid hormone-binding globulin
STAT	signal transducer and activator protein
TGF	transforming growth factor
TME	tumor microenvironment
TNF	tumor necrosis factor
TRAP	tartrate-resistant acid phosphatase
VCAM	vascular cell adhesion molecule
VDR	vitamin D 3 receptor
VEGF	vascular endothelial growth factor
Wnt	Wingless
Zeb-1	Zinc finger E-box-binding homeobox 1
